# Matrix Stiffness-Mediated DNA Methylation in Endothelial Cells

**DOI:** 10.1007/s12195-024-00836-9

**Published:** 2025-01-17

**Authors:** Paul V. Taufalele, Hannah K. Kirkham, Cynthia A. Reinhart-King

**Affiliations:** 1 Department of Biomedical Engineering, Vanderbilt University, Nashville, TN USA; 2 Bioengineering Department, Rice University, Houston, TX USA

**Keywords:** DNA methylation, Endothelial cells, Matrix stiffness, DNMT1

## Abstract

**Purpose:**

Altered tissue mechanics is a prominent feature of many pathological conditions including cancer. As such, much work has been dedicated to understanding how mechanical features of tissues contribute to pathogenesis. Interestingly, previous work has demonstrated that the tumor vasculature acquires pathological features in part due to enhanced tumor stiffening. To further understand how matrix mechanics may be translated into altered cell behavior and ultimately affect tumor vasculature function, we have investigated the effects of substrate stiffening on endothelial epigenetics. Specifically, we have focused on DNA methylation as recent work indicates DNA methylation in endothelial cells can contribute to aberrant behavior in a range of pathological conditions.

**Methods:**

Human umbilical vein endothelial cells (HUVECs) were seeded on stiff and compliant collagen-coated polyacrylamide gels and allowed to form monolayers over 5 days. DNA methylation was assessed via 5-methylcytosine ELISA assays and immunofluorescent staining. Gene expression was assessed via qPCR on RNA isolated from HUVECs seeded on collagen-coated polyacrylamide gels of varying stiffness.

**Results:**

Our work demonstrates that endothelial cells cultured on stiffer substrates exhibit lower levels of global DNA methylation relative to endothelial cells cultured on more compliant substrates. Interestingly, gene expression and DNA methylation dynamics suggest stiffness-mediated gene expression may play a role in establishing or maintaining differential DNA methylation levels in addition to enzyme activity. Additionally, we found that the process of passaging induced higher levels of global DNA methylation.

**Conclusions:**

Altogether, our results underscore the importance of considering cell culture substrate mechanics to preserve the epigenetic integrity of primary cells and obtain analyses that recapitulate the primary environment. Furthermore, these results serve as an important launching point for further work studying the intersection tissue mechanics and epigenetics under pathological conditions.

## Introduction

The vasculature system is a critical component of the tumor microenvironment. To grow, tumors must recruit blood vessels from pre-existing blood vessels through angiogenesis [1, 2]. However, tumor vasculature is characteristically unorganized, tortuous, and leaky [3]. Interestingly, physical cues such as extracellular matrix stiffness have been shown to play an important role in regulating endothelial cell behavior [4–6]. Furthermore, there is ample evidence demonstrating that many solid tumors are significantly stiffer than their normal tissue counterparts [7], in part due to excess matrix deposition or matrix cross-linking [8]. Our lab has previously demonstrated that several features of the tumor vasculature can be rescued by reducing matrix stiffening [9]. Specifically, reducing matrix stiffness decreases excessive angiogenesis and decreases vascular permeability [9]. As such targeting mechanotransduction and mechanical effects could be leveraged as a therapeutic strategy [10]. Here we seek to understand how mechanical properties may drive contribute to aberrant endothelial cell behavior in the tumor microenvironment.

The intersection of epigenetics with mechanobiology has been gaining interest [11, 12]. Epigenetics is the study of phenomena by which chromosomal regions are altered to register, signal, or perpetuate altered activity states [13]. One of the main epigenetic systems is DNA methylation [13]. In DNA methylation, a methyl group is covalently attached to a cytosine base in DNA [14]. In mammalian cells, this methylation occurs preferentially at ‘CG’ sequences [15]. DNA methylation traditionally has been shown to regulate gene expression by recruiting methyl binding proteins or directly inhibiting the binding of transcription factors [14]. Much attention has been placed onto the study of DNA methylation as it has found usage as a possible prognostic marker [16–20].

Interestingly, DNA methylation plays an important role in endothelial cells and disease progression. Recent work demonstrated that disturbed fluid flow induces changes in endothelial cell DNA methylation and gene expression which can contribute to atherosclerosis development [21–23]. Additional work has revealed endothelial cells exhibit decreased global DNA methylation levels during angiogenic programs, with corresponding specific correlations between changes in gene promoter methylation and RNA abundance [24]. Furthermore, Maishi et al. have shown that tumor endothelial cells, which are abnormal and exhibit pathological characteristics [25, 26], have altered levels of DNA methylation [27]. Specifically, tumor endothelial cells exhibited decreased DNA methylation at promoter region of biglycan resulting in higher expression [27].

As recent work in the field has demonstrated a link between mechanical cues and DNA methylation [12], we specifically focused on the effects of matrix stiffness on DNA methylation in endothelial cells. Utilizing collagen-coated polyacrylamide substrates, our data indicate endothelial cells cultured on increased stiffnesses display decreased levels of global DNA methylation, a decrease in the RNA abundance of DNMT1 which plays a role in propagating DNA methylation. Furthermore we find that global levels of DNA methylation decrease over time and the process of passaging increases global levels of DNA methylation.

## Results

### DNA Methylation Levels are Responsive to Substrate Stiffness

**Fig. 1:**
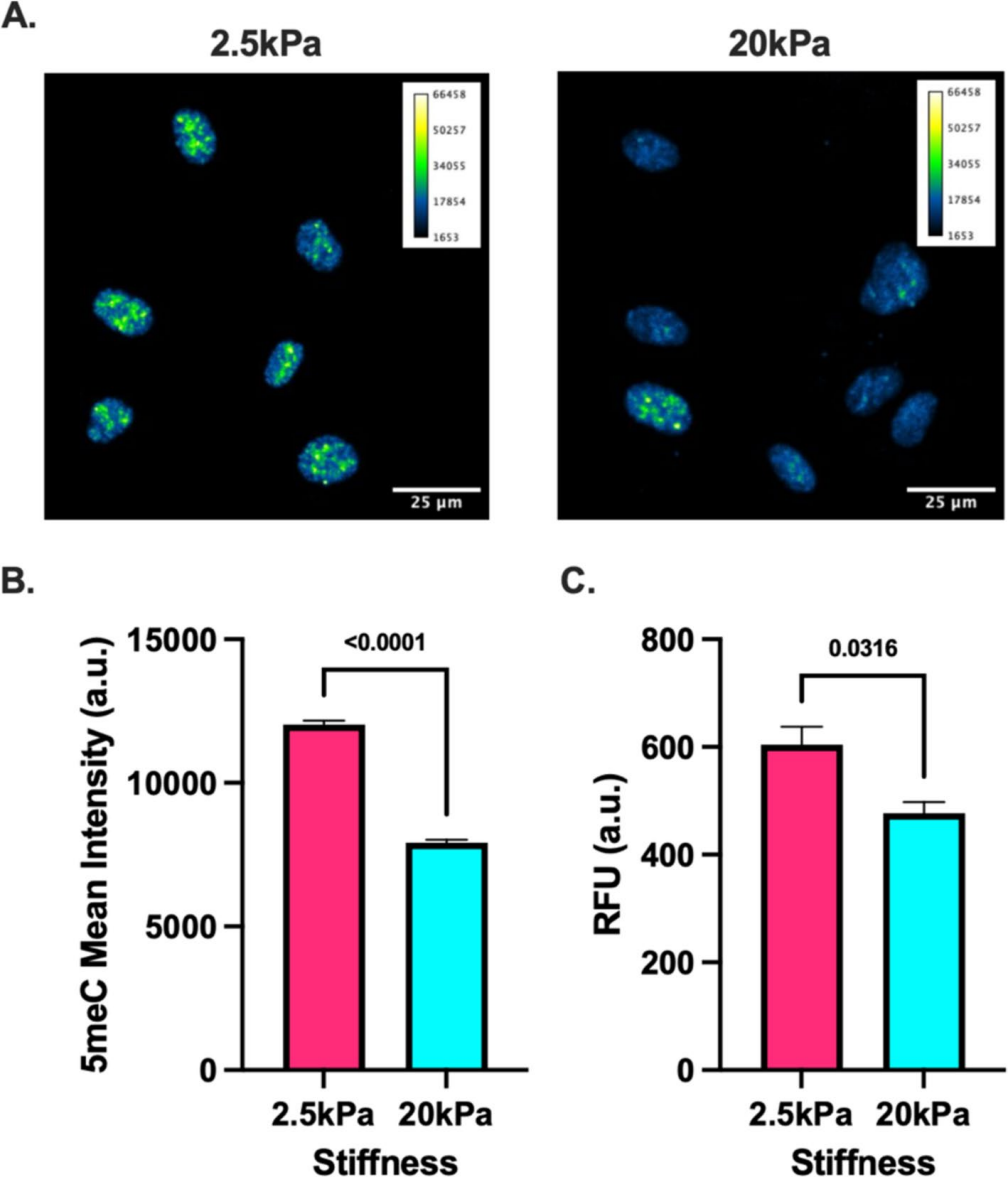
Stiffness mediated global DNA methylation levels. **A** Representative images of 5-methylcytosine immunofluorescent staining. **B** Quantification of 5-methylcytosine immunofluorescent staining in HUVECs cultured on PA gels. Mann Whitney test. *N* = 6, *n* = 1936–2039; **C** Quantification of 5-methylcytosine ELISA fluorescent intensity. Unpaired *t*-test. *N* = 3, *n* = 3

To investigate the effect of substrate stiffness on global DNA methylation levels in endothelial cells, we seeded Human Umbilical Vein Endothelial Cells (HUVECs) atop collagen-coated polyacrylamide (PA) gels of 2.5 and 20 kPa to mimic the range of heterogeneous stiffness observed in the tumor microenvironment [7, 28, 29]. After 5 days, global DNA methylation levels were assessed via immunofluorescent staining of fixed cells (Fig. 1A and B) and ELISA performed on isolated genomic DNA (Fig. 1C). Immunofluorescent staining of 5-methylcytosine revealed signal was predominantly localized to the nucleus (Fig. 1A). Interestingly, HUVECs seeded on stiffer substrates had significantly lower 5-methylcytosine signal in the nucleus (Fig. 1B). Furthermore, this result was confirmed by performing an ELISA on isolated DNA demonstrating significantly lower 5-methylcytosine levels in HUVECs cultured on stiffer substrates (Fig. 1C). Altogether, this data suggests that increased substrates stiffness induces lower levels of global DNA methylation.

### mRNA Abundance of DNMT1 is Reduced on Stiffer Substrates

**Fig. 2:**
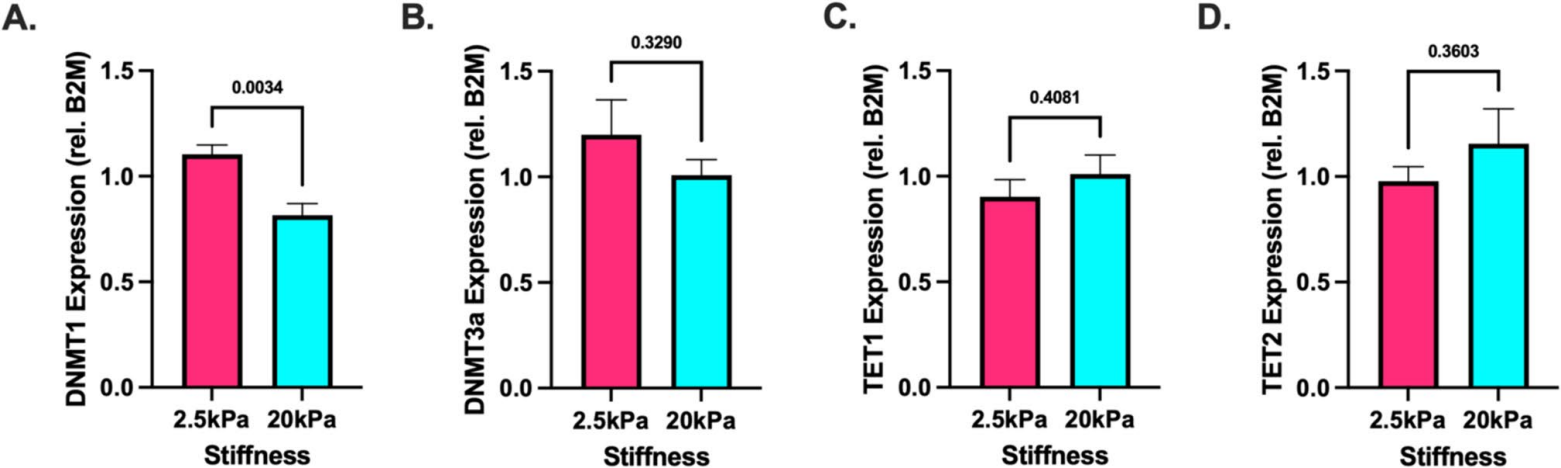
Stiffness-mediated gene expression. Quantification of **A** DNMT1, **B** DNMT3a, **C**TET1, and **D** TET2 RNA abundance measured by qPCR. Unpaired *t*-test. *N* = 4, *n* = 4–5

DNA methylation and demethylation can be accomplished by several known enzymes. To determine if substrate stiffness induces changes in overall abundance of these enzymes, we cultured HUVECs on compliant (2.5 kPa) and stiff (20 kPa) PA gels for 5 days and performed qPCR to measure RNA abundance. We performed qPCR on DNMT1, DNMT3a, DNMT3b, TET1, and TET2. Interestingly, qPCR revealed DNMT1 levels were significantly lower on stiffer substrates (Fig. 2). DNMT3a, TET1, and TET2 remained not significantly different (Fig. 2) and DNMT3b expression was not detected (data not shown). Altogether our results suggest that increased substrate stiffness induces lower levels of DNMT1 expression while the remaining enzymes involved in DNA methylation remain unchanged.

### Dynamics of Stiffness Responsive DNA Methylation

**Fig. 3:**
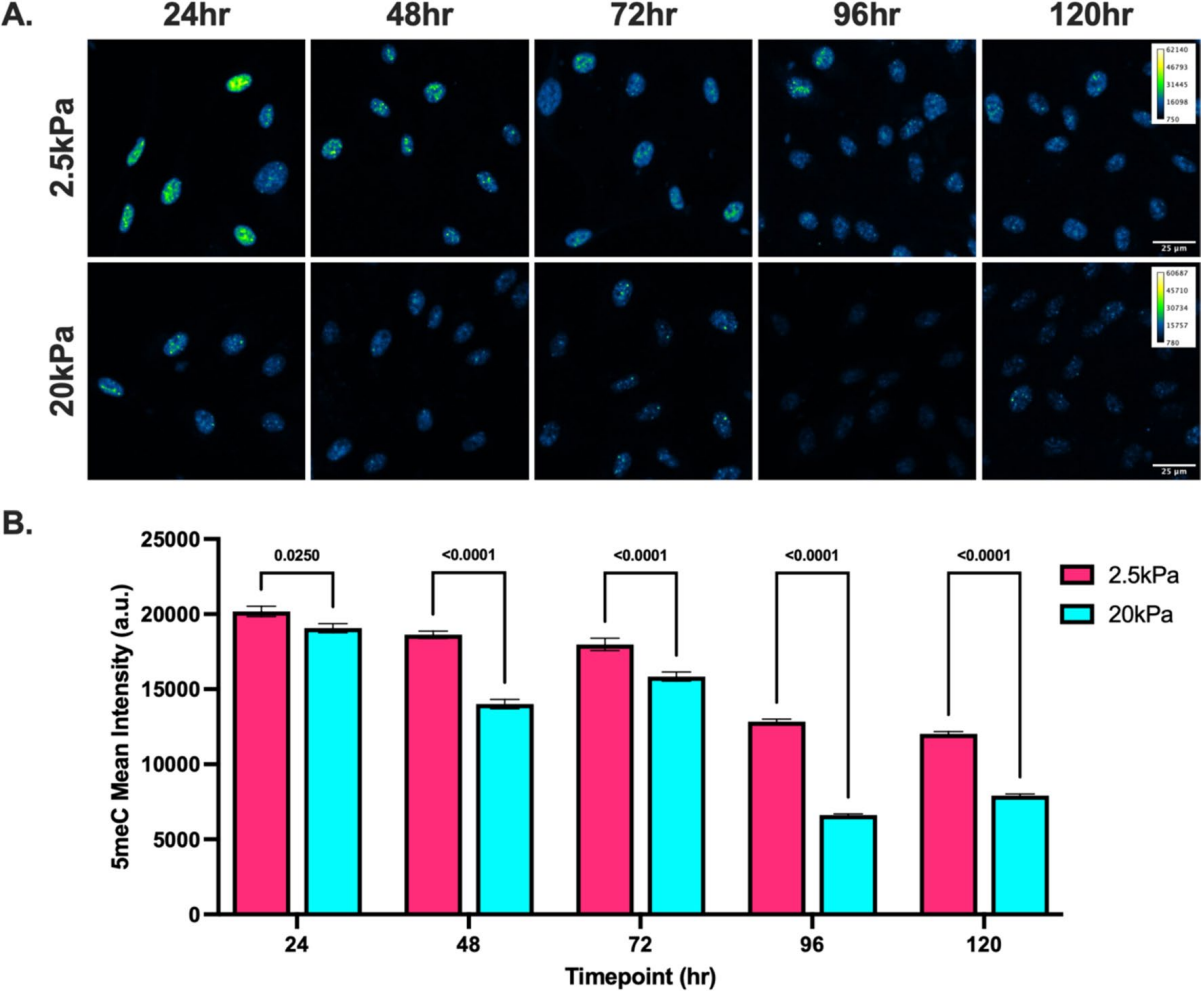
DNA methylation over time. **A** Representative 5-methylcytosine immunofluorescent staining of HUVECs cultured on PA gels over 5 days and **B** quantification of fluorescent intensity. Two-way ANOVA. *N* = 4–7, *n* = 523–2039

**Fig. 4:**
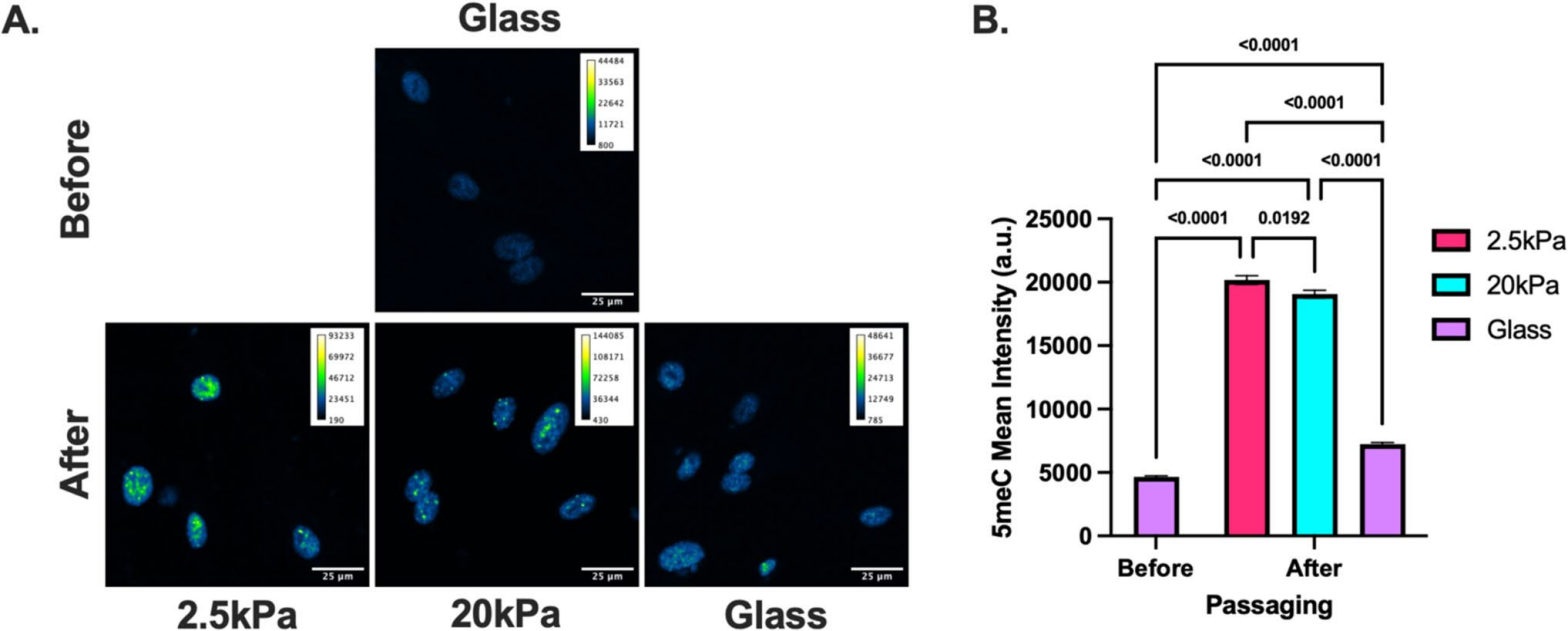
DNA methylation before and after passaging. **A** Representative 5-methylcytosine immunofluorescent staining of HUVECs seeded on PA gels or glass slides before or after passaging and **B** quantification of fluorescent intensity. Two-way ANOVA. *N* = 3–6, *n* = 339–596

To investigate the dynamics of DNA methylation in response to substrate stiffness, we seeded HUVECs on top of compliant (2.5 kPa) and stiff (20 kPa) PA gels and measured global DNA methylation levels via 5-methylcytosine staining every 24 h for 5 days (Fig. 3). Interestingly, our results demonstrate that DNA methylation levels are significantly lower on stiffer substrates after only 24 h of culture (Fig. 3). Furthermore, the data suggests that DNA methylation levels decreases over time in both stiffness conditions while the difference between stiff and compliant substrates remains significant. To assess the contribution of passaging to the changes in DNA methylation, we measured the DNA methylation levels of HUVECs cultured on glass slides prior to seeding on PA gels (Fig. 4). Additionally we added a glass slide condition at the 24 h time point to isolate specifically the effects of passaging on DNA methylation levels (Fig. 4). Interestingly, our results demonstrate that DNA methylation levels are significantly higher after passaging onto all three conditions compared to the HUVECs cultured on glass slides before passaging (Fig. 4). Altogether, these results indicate that DNA methylation levels may be responsive to substrate stiffness within 24 h of exposure and that the process of passaging cells increases DNA methylation levels.

## Discussion

Here, we demonstrate that global DNA methylation in endothelial cells is responsive to substrate stiffness. Specifically our data indicates increased substrate stiffness decreases global DNA methylation levels. Additionally, we show that levels of DNMT1, an enzyme responsible for methylating DNA, are congruent with global DNA methylation levels. Finally, our data suggests this difference in global DNA methylation level is evident as early as 24 h of exposure to substrates of varying stiffness and global DNA methylation levels decrease over time on both substrate stiffnesses while maintaining a significant difference compared to each other.

Recent studies investigating the effects matrix stiffness on DNA methylation have reported mixed results [30–34]. Two have demonstrated that increased substrate stiffness has no significant effect on global DNA methylation levels [31, 33], while one group has shown significant decreases [30] and two groups have shown significant increases [32, 34]. Other groups have focused on the methylation of a single promoter region in the genome, where some have demonstrated that increased substrate stiffness is associated with decreases in DNA methylation in a specific promoter region [35–37] while others have demonstrated a significant increase [38]. Interestingly, our work examines global DNA methylation levels (Fig. 1) and is in alignment with Xie et al. who show vascular smooth muscle cells decrease global DNA methylation on stiffer substrates [30]. There are several possibilities as to the discrepancy in the literature regarding the relationship between substrate stiffness and DNA methylation, including cell-type specific mechanisms. The studies cited above include the use of smooth muscle cells, various cancer cells, stem cells, epithelial cells, chondrocytes, and fibroblasts [30–38]. Different cell types vary not only in their compositions but in their functions. As such, much work has revealed the different ways in which different cell types respond to matrix stiffness [39, 40]. Thus, the differential change in global DNA methylation in response to substrate stiffness may be tied to the particular cell behaviors and internal mechanisms in each cell type. Furthermore, the range of stiffnesses used varies between studies. This is likely due to the particular context in which the cells were studied. We selected 2.5 kPa and 20 kPa to reflect the range of stiffnesses observed in the breast tumor microenvironment [7, 28, 29]. The mechanical properties of different tissues and pathologies vary and likely contribute to the selection of stiffnesses used in the studies [41]. Furthermore, cellular responses to stiffness may be non-linear [41] and biphasic [42–44]. Thus this suggests a limitation on extrapolating mechanoresponsive observations to different mechanical settings.

To investigate potential mechanisms underlying stiffness-mediated DNA methylation, we measured the RNA abundance to estimate the expression of the several key enzymes involved in DNA methylation. After culture on PA gels for 5 days, we measured the RNA abundance of several enzymes involved in methylating [45–47] and de-methylating DNA [48], and found DNMT1 significantly downregulated on stiffer substrates (Fig. 2). DNMT1 is a member of the DNA methyltransferase family of enzymes which can methylate DNA [45, 46]. Interestingly, DNMT1 is particularly involved in the maintenance of DNA methylation patterns through cell divisions [45–47], whereas its other family members DNMT3a and DNMT3b can carry out de novo methylation [45, 46]. Our data demonstrates that changes in DNA methylation occur as early as 24 h after seeding (Fig. 3). As HUVECs have doubling times of approximately 36 h [49], this stiffness-mediated DNA methylation may not be completely induced by DNMT1, as decreased propagation of DNA methylation would only be evident after cell division. Thus, it is likely that decreased DNMT1 levels on stiffer substrates contribute to the lower levels of global DNA methylation.

Interestingly, our data demonstrates that DNA methylation levels prior to seeding on PA gels are significantly lower than after seeding on both 2.5 kPa and 20 kPa stiffness PA gels (Fig. 3B). Furthermore, our data demonstrates that simply passaging cells induces a significant increase in DNA methylation levels (Fig. 3B). Intriguingly, a group has recently demonstrated that once cancer cells detach from the ECM, they exhibit increased global DNA methylation levels [50]. However, in our data, the increase solely from passaging is significantly less than the passaging onto both PA gel conditions. These results suggest that passaging cells increases their DNA methylation levels but cannot explain the increase seen on PA gels or the significant difference between the 2 PA gel conditions. DNA methylation levels are a balance of (1) de novo methylation via DNMT3a/b, (2) maintenance methylation via DNMT1, (3) replication-coupled passive methylation loss, and (4) active demethylation via TETs [51]. Furthermore, the balance of these processes could be effected by changes in the enzyme activity or expression levels. Since we observe differences in DNA methylation levels 24 h after passaging, which is likely prior to the division of most of the cells, we suspect that the initial passage mediated DNA methylation changes may occur due to either de novo methylation or active demethylation. The exact mechanisms remain to be elucidated.

Strikingly, our data is congruent with clinical data investigating DNA methylation in cancer. Specifically, hypermethylation at particular genomic loci is observed in tumors while globally hypomethylation is observed [52, 53]. Our results indicate that endothelial cells cultured on stiffer substrates exhibit decreased levels of DNA methylation. As matrix stiffening is associated with cancer progression, our data suggests that substrate stiffness may contribute to the observed DNA hypomethylation in tumors [9, 10, 52, 53]. As endothelial cells typically represent a small portion of the overall tumor microenvironment, further work is needed to determine if the remaining cell types in the tumor microenvironment follow a similar trend in stiffness mediated DNA hypomethylation [54, 55].

Although our time series data demonstrates that differences in DNA methylation between stiff and compliant conditions persist over a 5-day period, the levels in both conditions appear to decrease over time (Fig. 3). As noted above, we observed a subtle but significant increase in DNA methylation after passaging HUVECs and this is congruent with another observation by Nur et al. that cancer cells exhibit higher levels of global DNA methylation after detachment from the matrix [50]. Thus, it is possible that this anchorage dependent phenomena may be reversible after restoration of adhesion contacts. However, this likely can only attribute to a portion of the decrease, as DNA methylation levels are significantly higher after passage from glass onto PA gels compared to passage from glass onto glass. Another factor to consider is the confluence of the cell cultures. In this study, cells were first seeded at a sub-confluent level and allowed to grow to confluence over the 5 days. As endothelial cells undergo internal changes as they reach confluence, such as VE-cadherin phosphorylation [56], Weibel-Palade body formation [57], and cell cycle withdrawal [58], effects may be partially due to the cellular changes that occur during progression from sub-confluent to confluent monolayers.

Our work may be of interest to the field studying mechanical memory. Cells may be exposed to numerous mechanical forces and environments during development and disease [59]. For example, during metastasis, a cancer cells may traverse a heterogeneous primary tumor environment and to a secondary location [60, 61]. Additionally, tumors can progressively stiffen over time which exposes all cells residing in the tumor to more mechanical feedback [62]. Tumor angiogenesis entails the recruitment of vascular cells from surrounding healthy tissue into the tumor, in which the tumor tissue is typically stiffer than the healthy tissue counterpart [7]. Furthermore, time to initial cancer treatment in the United States after diagnosis ranges between 0 and 50 days [63]. As many drugs in development are targeting tissue stiffening, it will be important to understand how cells will respond to new mechanical properties or mechanical signaling after initiation of drug treatment [10]. Importantly, epigenetic regulation has been demonstrated to play a key role in mechanical memory. Particularly, nuclear deformation and actomyosin contractility can induce epigenetic effects such as histone acetylation, histone methylation, and DNA methylation [64]. Our work indicates that global DNA methylation levels are significantly altered by substrate stiffness and the effects emerge after 24 h and persist at least 120 h. Importantly, this demonstrates that substrate mechanics can induce epigenetic effects. As the majority of cell culture platforms vary from the mechanical environment of primary tissue, our work highlights the important need to consider mechanical properties of cell culture platforms to ensure in vitro results faithfully recapitulate in vivo phenomena. Future work should address the particular loci where methylation events occur due to differences in effects based on the particular location of DNA methylation [65]. Furthermore, links between altered DNA methylation states and functional consequences in gene expression or cell behavior remain a prime area of interest due to the development and implication of drugs targeting DNA methylation and other epigenetic marks [66].

## Methods

### Cell Culture

Human Umbilical Vein Endothelial cells (HUVECs) were purchased from Lonza [Lonza; C2519A]. HUVECs were maintained in Endothelial Cell Growth Medium-2 BulletKits (EGM-2) [Lonza; CC-3162] with 1% penicillin-streptomycin [Gibco; 15140122] and HUVECs cultured on PA gels or glass slides were cultured in M199 medium [Gibco; 11150067] supplemented with Endothelial Cell Growth Medium SingleQuots Supplements [CC-4133] and 1% penicillin-streptomycin. HUVECs were maintained at 37C and 5% CO_2_ incubators and utilized up to passage 5 for all experiments.

### Polyacrylamide Gel Preparation

Polyacrylamide gels (PA gels) were fabricated as previously described. In brief, glass slides were activated by plasma treatment [Harrick Plasma; Plasma Cleaner PDC-001] for 2 min, incubated in 1% polyethyleneimine [Sigma-Aldrich; P3143] for 10 min, washed 3 times in DI water, incubated in 0.1% glutaraldehyde [Sigma-Aldrich; G7776] in phosphate buffered saline without calcium or magnesium (PBS) [Gibco; 14200166 (10X stock)], washed 3 times in DI water, and allowed to air dry overnight. To generate PA gels of varying stiffness, the ratio of acrylamide [BioRad; 1610140] to bis-acrylamide [BioRad; 1610142] was varied in solution containing 70 mM HEPES pH6 and 0.1% v/v TEMED. For 2.5 kPa and 20 kPa PA gels, the ratio of acrylamide to bis-acrylamide was 5%:0.1% and 12%:0.19%, respectively. The pH of the PA gel mixes were adjusted to pH6 using 2 M HCl and degassed prior to polymerization with 10% ammonium persulfate [BioRad; 1610700]. The gels were functionalized with N-6-((acryloyl)amido)hexanoic acid (N6) (synthesized in-house) to allow covalent attachment of 0.1 mg/ml rat tail type I collagen [Corning; 354236] in 50 mM HEPES pH8. Excess N6 was neutralized with 1:1000 ethanolamine in 50 mM HEPES pH8. Polymerized gels were incubated in PBS supplemented with 4% penicillin-streptomycin overnight and exposed to UV light for 1 h in a biosafety cabinet prior to cell seeding.

### Immunohistochemistry

Prior to fixing, samples were briefly washed 2 × with 1X PBS. Samples were fixed in 3.2% PFA in 1X PBS for 5 min at room temperature. After fixation, samples were washed 3X with 1X PBS, permeabilized in 0.1% Triton-X100 [JT Baker; X198-07 (Octyl Phenol Ethoxylate)] in 1X PBS for 5 min and then washed 3 × with 0.02% tween 20 [Fisher Scientific; BP337-100 (Polysorbate 20)] in 1X PBS. For methylated cytosine antigen retrieval, samples were incubated in 2 M HCl in PBS for 30 min at 37C. Directly after, samples were neutralized with 0.1 M Tris-HCl pH8 for 5 min at room temperature. Samples were washed 3X in 0.02% tween 20 in 1X PBS and then placed in blocking solution for 1 h at room temperature. Blocking solution was composed of 10% donkey serum [Sigma-Aldrich; S30-100ML] and 10% fetal bovine serum [Corning; 35010CV] in 0.02% tween 20 in 1X PBS. After blocking, samples were incubated with primary antibodies in blocking solution overnight at 4C. To measure DNA methylation, primary antibodies against 5-methylcytosine were used. OptimAb Anti-5-methylcytosine (33D3) [Eurogentec; BI-MECY-0100 (mouse)] was used at a dilution of 1:450 for staining HUVECs and recombinant anti-5methylcytosine (RM231) [Abcam; ab214727 (rabbit)] was used at a dilution of 1:450 for staining mouse tumor sections. After primary staining overnight, samples were washed 3 × with 0.02% tween 20 in 1X PBS and placed in secondary antibodies and DAPI for 1 h at room temperature. For secondary staining 1:100 dilution of donkey anti-mouse secondary antibodies were used and 1:300 dilution of DAPI was used. After secondary staining, samples were washed 2 × with 0.02% tween 20 in 1X PBS and 2 × in 1X PBS before mounting on glass slides in vectashield antifade mounting medium [Vector Laboratories; H100010] and imaged on an LSM700 confocal microscope.

### Confocal Microscopy

Immunofluorescence stained samples were visualized using a Zeiss Axio Examiner.Z1 equipped with a LSM700 confocal module using a W Plan-Apochromat 20 × /1.0 N.A. water immersion objective operated by Zen 2010 software. For each image, 3 z-stacks were captured at 3.785 micron intervals. Images were captured with a size of 1024 × 1024 pixels with a resolution of 3.1991 pixels per micron.

### Image Analysis

Images were analyzed in Fiji (https://imagej.net/software/fiji/) with the aid of custom scripts. In brief, z-stacks were combined using SUM projections and a threshold was used on the channel containing signal from DNA methylation. The ‘analyze particles’ function was utilized to obtain ROI’s for every nucleus within the field of view. Quality control was performed manually to ensure debris or noise was not included as an ROI and mean intensities were measured for every ROI. A simple background subtraction was performed to obtain a mean fluorescent intensity.

### DNA Isolation and Methyl-Cytosine Quantification

PA gels with HUVECs were turned over onto a droplet of TRIZOL [Invitrogen; 155966026] and incubated at room temperature for 5 min. The PA gels were rinsed with the TRIZOL carefully by pipette and the TRIZOL solution containing the cell material was transferred into a microcentrifuge tube and allowed to incubate at room temperature for another 5 min. Chloroform was added to the TRIZOL per manufacturer’s instructions (0.2 mL chloroform for every 1 mL TRIZOL) and vigorously shaken. Samples were centrifuged at 4 °C for 30 min at 12,000 × g. The clear aqueous phase at the top was removed for subsequent RNA isolation. 100% ethanol was added to the remaining organic and interphase to precipitate the DNA. Samples were centrifuged at 4 °C for 5 min at 4000 × g to pellet the DNA. The pellet was washed twice with 0.1 M sodium citrate in 10% ethanol, pH 8.5 for 30 min. Then the pellet wash washed with 75% ethanol before allowing to dry and resuspended in 8 mM NaOH. To further purify DNA, we utilized ethanol precipitation as described previously [67]. In brief, two volumes of ice-cold ethanol and 2 mM ammonium acetate were added to the resuspended DNA and stored overnight at 4 °C. DNA was recovered by centrifugation at 4 °C at max speed for 10 min. The pellets were washed 2 × in 70% ethanol and resuspended in 8 mM NaOH. DNA concentration and purity was measured via nanodrop [Mettler Toledo; UV5 Nano]. To quantify methylcytosine levels in isolated genomic DNA, we utilized the Methylated DNA Quantification Kit (Fluorometric) [Abcam; ab117129] as per manufacturer’s instructions.

### RNA Isolation

A combination of TRIZOL and RNeasy Micro Kits [Qiagen; 74004] were used to isolate RNA from HUVECs cultured on top of PA gels. In brief, the clear aqueous phase from the DNA isolation section above was added to 0.5 mL of 70% ethanol and mixed by pipetting. This mixture was then applied to the RNeasy columns by centrifugation at 10,000 × g for 30 s. The samples were washed with 0.7 mL of RW1 buffer followed by two washes with RPE buffer. Samples were centrifuged without any wash buffer to allow for drying and then eluted in 35 µL of DNase-RNase free water. RNA concentration and purity was measured via nanodrop.

### RT-qPCR

To perform reverse-transcription quantitative PCR, we utilized the iScript cDNA synthesis kits [BioRad; 1708890] and iQ SYBR green supermix [BioRad; 1708882] according to manufacturer’s instructions. DNA oligo primers were ordered from Sigma Genosys through the Vanderbilt Molecular Biology Core. The following sequences were used for qPCR: DNMT1: fwd:GTCTGCTCCTGCGTGGAAG and rev: TTGGTGACGGTTGTGCTGAA. DNMT3a fwd: TCTTCGTTGGAGGAATGTGC and rev: AAAAGCACCTGCAGCAGTTG. DNMT3b fwd: AATAAGTCGAAGGTGCGTCG and rev: TTCATCCCCTCGGTCTTTGC. TET1 fwd: AATGGAAGCACTGTGGTTTG and rev: ACATGGAGCTGCTCATCTTG. TET2: GTGAGATCACTCACCCATCG and rev: CAGCATCATCAGCATCACAG. B2M: CACCCCCACTGAAAAAGATGAG and rev: CCTCCATGATGCTGCTTACATG. B2M served as housekeeping control gene. Samples were run on a Biorad thermocycler [CFX96 Real-Time System] and analyzed via the Biorad CFX Maestro Software.

### Statistical Analysis

Statistical analysis was performed using GraphPad Prism 9.0 [GraphPad Software; La Jolla, CA, USA]. The non-parametric unpaired Mann-Whitney test was performed on image analysis results from immunofluorescence staining of DNA methylation. Unpaired two-tailed t-tests with Welch’s correction were performed on ELISA and qPCR results. An ordinary two-way ANOVA with Sidak’s multiple comparison test was performed on timeseries methylation data. ‘N’ represents the number of independent biological replicates and ‘n’ represents the number of measurements made.

## Citation Diversity Statement

Racial, ethnic, and gender imbalances have been identified in the citation practice in the sciences [68, 69]. As such, we sought to proactively confront implicit bias by integrating diversity and inclusion principles towards our reference list.

## Data Availability

Per NIH guidelines, the data is available upon request.
